# Protospacer Adjacent Motif (PAM)-Distal Sequences Engage CRISPR Cas9 DNA Target Cleavage

**DOI:** 10.1371/journal.pone.0109213

**Published:** 2014-10-02

**Authors:** Regina Cencic, Hisashi Miura, Abba Malina, Francis Robert, Sylvain Ethier, T. Martin Schmeing, Josée Dostie, Jerry Pelletier

**Affiliations:** 1 Department of Biochemistry, McGill University, Montreal, Québec, Canada; 2 The Rosalind and Morris Goodman Cancer Research Center, McGill University, Montreal, Québec, Canada; 3 Department of Oncology, McGill University, Montreal, Québec, Canada; Osaka University, Japan

## Abstract

The clustered regularly interspaced short palindromic repeat (CRISPR)-associated enzyme Cas9 is an RNA-guided nuclease that has been widely adapted for genome editing in eukaryotic cells. However, the *in vivo* target specificity of Cas9 is poorly understood and most studies rely on *in silico* predictions to define the potential off-target editing spectrum. Using chromatin immunoprecipitation followed by sequencing (ChIP-seq), we delineate the genome-wide binding panorama of catalytically inactive Cas9 directed by two different single guide (sg) RNAs targeting the *Trp53* locus. Cas9:sgRNA complexes are able to load onto multiple sites with short seed regions adjacent to ^5′^NGG^3′^ protospacer adjacent motifs (PAM). Yet among 43 ChIP-seq sites harboring seed regions analyzed for mutational status, we find editing only at the intended on-target locus and one off-target site. *In vitro* analysis of target site recognition revealed that interactions between the 5′ end of the guide and PAM-distal target sequences are necessary to efficiently engage Cas9 nucleolytic activity, providing an explanation for why off-target editing is significantly lower than expected from ChIP-seq data.

## Introduction

The *Streptococcus pyogenes* CRISPR (clustered, regularly interspaced short palindromic repeat) endonuclease Cas9 (CRISPR-associated), in conjunction with a bifunctional single guide (sg) RNA that binds Cas9 and targets a ∼20 nucleotide (nt) genomic address via base complementarity, has become the tool of choice for a number of precise genome editing applications *ex vivo* [1]–[8] and *in vivo* [9]. Target recognition by Cas9:sgRNA complexes involves sampling of genomic DNA for the presence of protospacer adjacent motifs (PAM) (usually ^5′^NGG^3′^) [2], [10] followed by RNA-DNA heteroduplex initiation proceeding from the PAM towards the distal end of the target sequence [11]. Target specificity is not well understood and off-target sites are generally deduced from alignment algorithms using a seed sequence of 11–13 nts. Some studies indicate a very high degree of specificity [12]–[14] while others intimate far more promiscuous editing activity [15]–[19]. However, two recent ChIP-seq studies have reported that Cas9 binds a multitude of off-target sites, with some harboring seed sequences as short as 5 nts [20], [21]. Strikingly, virtually no editing activity was detected at a large number of these sites when probed for the presence of mutations, suggesting that target binding *per se* is insufficient to trigger DNA cleavage [20], [21]. Herein, we have investigated the off-target binding spectrum of sgRNAs targeting p53 using ChIP-seq. *In vitro* characterization of Cas9 binding and cleavage revealed variations in efficiencies dictated by interactions between the 5′ end of the sgRNA guide region and PAM distal target sequences. All-in-one retroviral delivery vectors co-expressing two sgRNAs and a previously described Cas9(D10A) nickase mitigated off-target editing. Our results indicate that the sequence requirements for Cas9 DNA binding are different from those for catalytic activity – with PAM-distal target and 5′ sgRNA interactions being critical for DNA cleavage.

## Results

**Figure 1:**
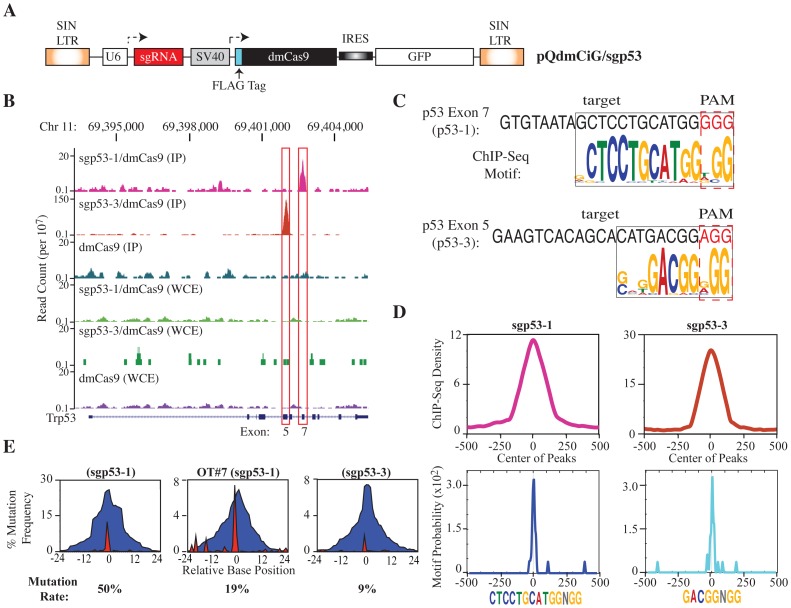
Genome-wide binding of sgp53/dmCas9. **A.** Schematic representation of retroviral vector design expressing sgp53-1 or sgp53-3, dmCas9, and GFP. **B**. Genomic tracks displaying ChIP-seq and WCE-seq data from sgp53-1/dmCas9-, sgp53-3/dmCas9-, and dmCas9-infected cells across a ∼12 kbp region spanning p53. The RefSeq gene track for *Trp53* is shown below the profiles. **C**. Identification of enriched motifs in sgp53-1/dmCas9 and sgp53-3/dmCas9 ChIP samples. The p53 exon target site and flanking PAM (red) are indicated. The sequence logo depicts the nucleotide distributions of overrepresented binding sites found by MEME-ChIP analysis in segments targeted specifically by sgp53-1/dmCas9 (25 sites, p-value 1.4×10^−20^) and sgp53-3/dmCas9 (24 sites, p value 3.2×10^−15^). **D**. Top: ChIP-enriched sequence read density. Bottom: Distribution of seed motif adjacent to a PAM within ∼500 bp of peak summits. **E**. Location and mutation frequency across 48 nucleotides centered from the 3^rd^ nucleotide of the seed sequence upstream of the PAM (set at 0) for sgp53-1, OT#7, and sgp53-3 target sites. Blue indicates deletion and red indicates insertions. The percentage of read counts harboring mutations is indicated below the panel.

We previously reported targeting of the *Trp53* locus via linked co-transduction of Cas9 and an exon 7 targeting sgRNA for modeling *in vivo* cancer initiating lesions [22]. In this study, we documented off-target editing events at only one of nine genomic loci chosen based on the presence of a 13 base pair (bp) perfect match to the 3′ end of the guide sequence [22]. Albeit heuristic and expedient, this target selection approach based on computational sequence alignment is limited in scope and inevitably biased. We undertook a series of ChIP-seq experiments with Cas9 as an unbiased, whole genome, and *in vivo* approach to assess the spectrum of potential off-target cleavage sites. Towards this end, we transduced *Arf^−/−^* MEFs, a cell line previously characterized to contain an intact TRP53 locus [29], with a FLAG-tagged, catalytically inactive double mutant (dm) Cas9 (D10A/H840A) without or in combination with two previously characterized and functional sgRNAs targeting *Trp53* exons 5 or 7 (Figure 1A) [22]. DNA obtained from α-FLAG immunoprecipitates from these and mock-infected cells was sequenced (Table 1). For comparison, we also sequenced the total input DNA from all dmCas9 IPs (WCE DNA) and mock-infected Arf^−/−^ cells (Table 1). Analysis of ChIP-seq data from sgp53-1/dmCas9 and sgp53-3/dmCas9 identified 144 and 44 enriched and unique peaks, respectively, which included the expected enrichment of the corresponding targeted exons (Figure 1B and Table S1). *De novo* motif discovery on these sets yielded predicted elements with striking similarity to the seed sequences adjacent to a PAM. For the sgp53-1 series, the motif matched the 11 most 3′-nts of the target (^5′^ CTCCTGCATGGNGG ^3′^), while analysis from the sgp53-3 series yielded a motif that matched only the 5 most 3′-nts of the target and a PAM (^5′^ GACGGNGG ^3′^) (Figure 1C). Each motif mapped to 13% and 54% of the enriched peaks for sgp53-1 or sgp53-3 dmCas9-bound samples, respectively (Table S1, yellow highlight). The ChIP-enriched reads showed a non-random, summit distribution spanning ∼150 bps (Figure 1D, top) and the identified motifs were centrally located within the resident peaks (Figure 1D, bottom). For sgp53-1, many of our previously characterized off-target sites were also highly enriched with a centrally localized motif (OT#4, OT#7, OT#3, OT#8, and OT#1) (Table S1, highlighted in red) [22]. We also identified several new loci, 19 for sgp53-1 and three for sgp53-3, which collectively represent ∼18% and 50% of all predicted genomic sites that conform to an 11 bp seed motif, as deduced from a genome-wide alignment search (Table S2, highlighted in yellow). The much shorter motif derived from the sgp53-3/dmCas9 ChIP-seq sample, although at odds with the 11–13 nt seed sequence that is generally considered when predicting off-target sites, is in line with recently published ChIP-seq data performed with dmCas9 using different sgRNAs [20], [21].

**Table 1:** Number and Percentage of Total, Aligned, Duplicate, and Processed Reads Obtained from Chromatin Immunoprecipitates or Whole Cell Extracts (WCE) of Arf^−/−^ MEFs infected with pQdmCiG, pQdmCiG/sgp53-1, or pQdmCiG/sgp53-3.

Sample ID	Read Count	Unique Alignments	Multiple Alignments	No Alignments	Duplication Rate	Processed Reads	Processed Read Counts*
IP (sgp53-1/dmCas9)	20,349,675	9,996,563 (49.1%)	9,513,057 (46.7%)	840,055 (4.2%)	27%	60%	12,292,686
IP (sgp53-3/dmCas9)	9,151,463	4,636,690 (50.7%)	4,321,439 (47.2%)	193,334 (2.1%)	15%	72%	6,632,003
IP (dmCas9)	34,153,745	16,419,713 (48.1%)	15,372,527 (45%)	2,361,505 (6.9%)	50%	39%	13,207,154
IP (WT)	17,833,001	9,143,908 (51.3%)	8,313,527 (46.6%)	375,566 (2.1%)	13%	75%	13,400,263
WCE (sgp53-1/dmCas9)	32,832,902	16,842,471 (51.3%)	15,514,245 (47.2%)	476,186 (1.4%)	19%	71%	23,262,915
WCE (sgp53-3/dmCas9)	4,284,130	2,132,290 (49.8%)	1,966,892 (45.9%)	184,948 (4.3%)	12%	74%	3,153,672
WCE (dmCas9)	27,255,805	13,950,188 (51.1%)	12,646,386 (46.4%)	659,231 (2.4%)	14%	75%	20,354,775
WCE (WT)	5,589,669	2,784,010 (49.8%)	2,567,753 (45.9%)	237,906 (4.3%)	12%	73%	4,108,236

*After mapping and removal of duplicates.

We next tested whether the predicted, unique and enriched ChIP-seq peaks corresponded to a genome-editing event. The *Arf^−/−^* MEF parental cell line was infected with virus expressing Cas9 and either sgp53-1 or sgp53-3 sgRNAs and enriched for GFP^+^ cells via FACS. Importantly, all infected cells expressed similar amounts of Cas9 and dmCas9 (Figure S1). For sgp53-1, we generated 34 unique primer pairs that probed: (i) p53 exon 7 target, (ii) the 5 previously predicted off-target sites that were re-identified in the ChIP-seq experiment (OT#4, OT#7, OT#3, OT#8, and OT#1), (iii) the 19 loci newly identified by ChIP-seq and harboring the 11 nt seed motif, and (iv) the top 9 computationally predicted genomic regions not identified in the ChIP experiment but containing a perfect seed motif and the least amount of mismatches compared to the 20 nt guide sequence (Table S3). For p53-3, we designed 9 primer pairs that probed the p53 exon 5 target site and 8 loci identified by ChIP-seq (Table S3). Of the 43 sites assessed, only *Trp53* exons 5 and 7 and the previously identified OT#7 site (which has only 2 mismatches relative to *Trp53*) contained mutations (Figure 1E), indicating virtually no correlation between dmCas9 binding sites identified by ChIP-seq and actual Cas9-modified off-target sites.

**Figure 2:**
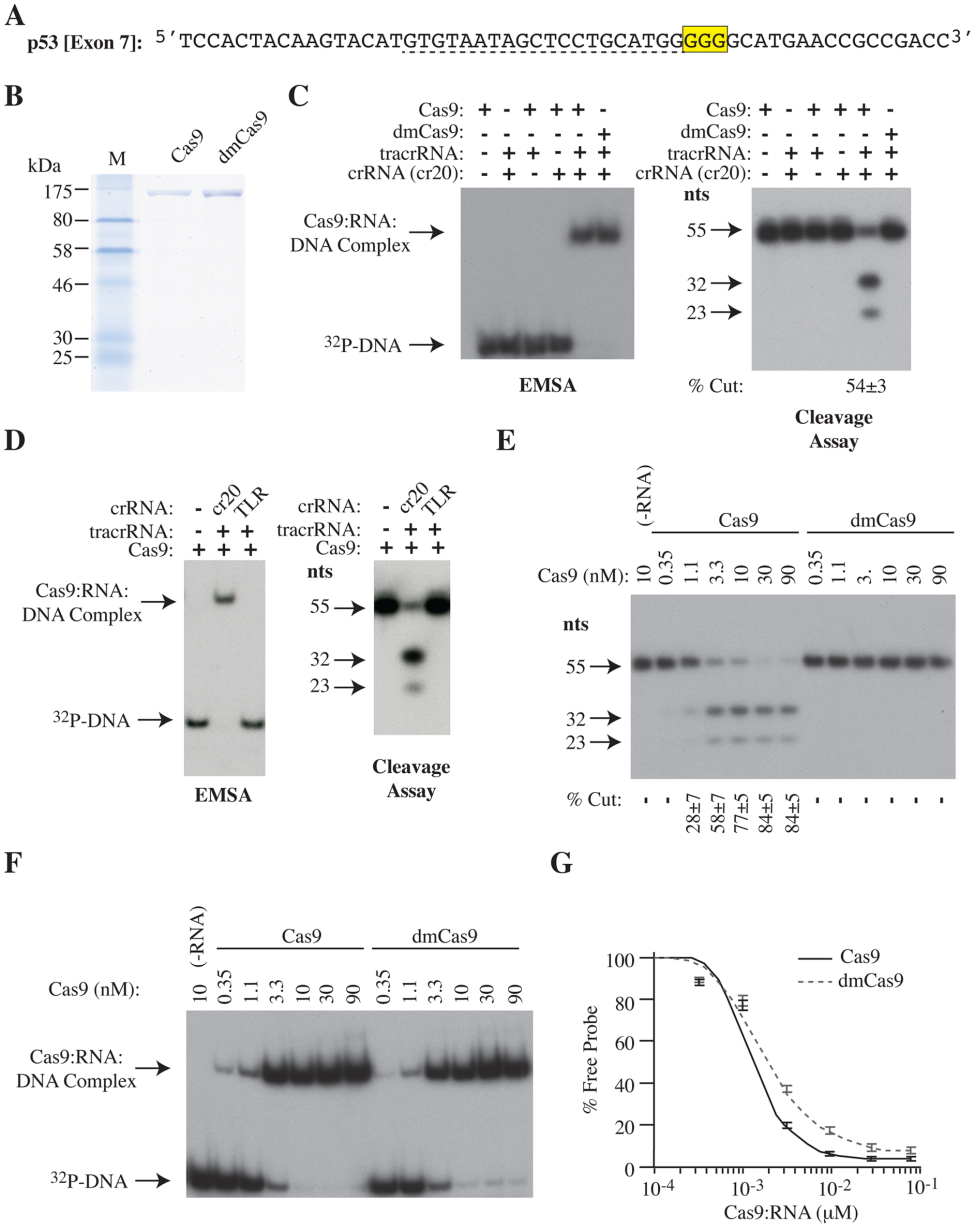
*In vitro* DNA binding and cleavage properties of recombinant Cas9 and dmCas9 to the p53-1 target site. **A.** Nucleotide sequence of oligonucleotide probes harboring the p53 exon 7-target site. The PAM motif is highlighted in yellow. The 20 nucleotide guide target is underlined by a dash line. **B**. Coomassie-stained SDS-PAGE of purified recombinant Cas9 and dmCas9 protein. **C**. *In vitro* binding to, and cleavage of, p53 [Exon 7] by Cas9. The presence or absence of Cas9, dmCas9, tracrRNA, and crRNA (harboring a guide sequence to *Trp53* exon 7; cr20: ^5′^ GUGUAAUAGCUCCUGCAUGG ^3′^) is indicated above the panels. Left panel: EMSA resolved on a 5% native polyacrylamide gel. Right panel: Visualization of p53 [Exon 7] cleavage products by Cas9/tracr/crRNA resolved on a 10% polyacrylamide/8M urea gel. crRNA, CRISPR RNA; tracrRNA, trans-activating crRNA. **D**. Specificity of binding to, and cleavage of p53 [Exon 7]. EMSA and cleavage assays were performed with a crRNA targeting *Trp53* exon 7 (p53-1) or a neutral control (TLR: ^5′^ GAGCAGCGUCUUCGAGAGUG ^3′^). **E**. Cleavage of p53 [Exon 7] by the indicated concentrations of Cas9. The “-RNA” lane indicates the absence of both crRNA and tracrRNA. Quantification is shown below the panel in the reaction mix. -, below limit of detection. n = 3±SD. **F**. EMSA with the indicated concentrations of Cas9 or dmCas9 to p53 [Exon 7]. The “-RNA” lane indicates the absence of both p53-1crRNA and tracrRNA. **G**. Quantification of EMSA of Cas9 and dmCas9 binding to p53 [Exon 7] in the presence of tracrRNA and crRNA (cr20). Quantitations were performed on a Typhoon Trio Variable Mode Imager with a Fuji imaging screen. n = 3±SD.

The lack of predictive power of ChIP-seq in establishing *bona fide* CRISPR-targeted altered sites suggested potential DNA sequence-specific driven differences between binding and cleavage events of an sgRNA-engaged Cas9. To better understand this discrepancy, we analyzed more closely the requirement for the seed motif mediating these differences. We targeted a 55 bp oligonucleotide duplex harboring the sgp53-1 exon 7 homologous target site with its native PAM (Figure 2A) using purified recombinant wild-type Cas9 or dmCas9 (Figure 2B). As expected, both proteins were able to form complexes with their cognate substrate in the presence of crRNA and tracrRNA but only Cas9 was able to induce cleavage (Figure 2C). These complexes were the result of specific crRNA:DNA heteroduplex formation, since Cas9 was unable to bind to the *Trp53* [Exon 7] probe when the crRNA was replaced with one that targets an unrelated sequence (Figure 2D, TLR crRNA). Titration of Cas9 and dmCas9 in complex with crRNA and tracrRNA revealed that both had similar equilibrium dissociation constants (K_d_) for the *Trp53* target substrate (K_d_[Cas9] = 1.5 nM; K_d_[dmCas9] = 2 nM) (Figure 2E–G and Table 2), similar to a previously reported value of 0.5 nM for Cas9 [11]. These results indicate that the discrepancy between dmCas9 binding site identification and Cas9 mutational spectrum *in vivo* is unlikely a consequence of significantly different DNA binding affinities between the two proteins.

**Table 2:** Binding Data for Cas9, dmCas9, and crRNA Substrates.

Cas9:crRNA	DNA Target	K_d_(nM)^a^	K_d, rel_ b
Cas9:cr20	p53[Exon 7]	1.5±0.1	-
dmCas9:cr20	p53[Exon 7]	2.0±0.2	1.3
Cas9:cr15	p53[Exon 7]	8.8±1.8	5.9

a Reported as the average and standard error of the mean from 2–3 independent experiments.

b Calculated by dividing each K_d_ value by the K_d_ for Cas9:cr20:tracrRNA:p53[Exon 7].

**Figure 3:**
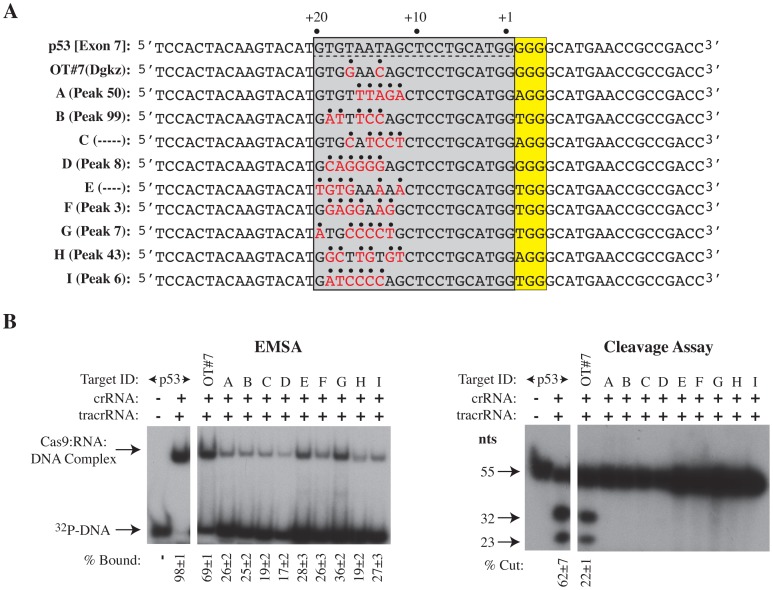
Assessment of DNA binding and cleavage by Cas9 to sites identified by ChIP-Seq or *in silico* prediction. **A.** Sequence of DNA probes harboring the wt p53 target (underlined) and adjacent PAM (highlighted in yellow) sequence or target sequences (from ChIP-seq or *in silico* prediction) harboring an 11 nt seed+PAM. Nucleotide differences relative to the wt p53 target site are highlighted in red with an overhead dot. The complete sequence of the 5′ and 3′ regions (not highlighted) of the oligonucleotides were maintained constant in all probes and originate from the p53 locus. Note that probes for C and E do not have an associated peak number since they are bioinformatically predicted and were not identified by ChIP-Seq. **B**. Left Panel. Assessment of Cas9 binding to oligonucleotide probes shown in Panel A. Reactions were resolved on a 5% native polyacrylamide gel. Right Panel. Cleavage reactions with DNA probes shown in Panel A. Reactions were resolved on a 10% polyacrylamide/8 M urea gel. Quantifications were performed on a Typhoon Trio Variable Mode Imager with a Fuji imaging screen. n = 3±SD. All samples were analyzed on the same gel - controls are juxtaposed adjacent to the experimentals for clarity.

We interpret the results from the ChIP-Seq experiment to indicate that extensive perturbation beyond the seed region affects Cas9 target cleavage but not complex formation. This was directly tested using probes that contained either the p53 [Exon 7] target site, the most highly conserved binding sites identified from the sgp53-1/Cas9 ChIP-seq experiment (OT#7, A, B, D, F, G, H, and I) or predicted binding sites (C and E) not present in the ChIP-Seq data (Figure 3A). All sites were capable of forming Cas9:RNA:DNA complexes albeit to varying efficiencies, with the most prominent complexes obtained with probes harboring the p53 [Exon7] site and OT#7, yet cleavage occurred only at p53 [Exon 7] and OT#7 (Figure 3B). Noticeably, of the ChIP-seq targets tested in this assay, OT#7 had the least number of mismatches with p53-1 crRNA (positions 14 and 17). We were concerned that our inability to detect cleavage at the off-target sites was simply an indirect consequence of reduced binding of Cas9 to these sites. To directly address this, we isolated Cas9:RNA:DNA complexes for several of the tested sites directly from the EMSA gel, followed by re-analysis of the radiolabelled DNA products on a denaturing polyacrylamide gel. The results indicate that endonucleolytic cleavage was detected only with the p53 [Exon 7] and OT#7 probes (Fig. S2). The concordance between our *in vitro* assays and the *in vivo* ChIP data indicated that Cas9/sgRNA complexes can bind to many sites with significant nucleotide divergence to the intended target template, but that extensive complementarity between the 5′ crRNA and PAM distal target sequence dictates cleavage.

**Figure 4:**
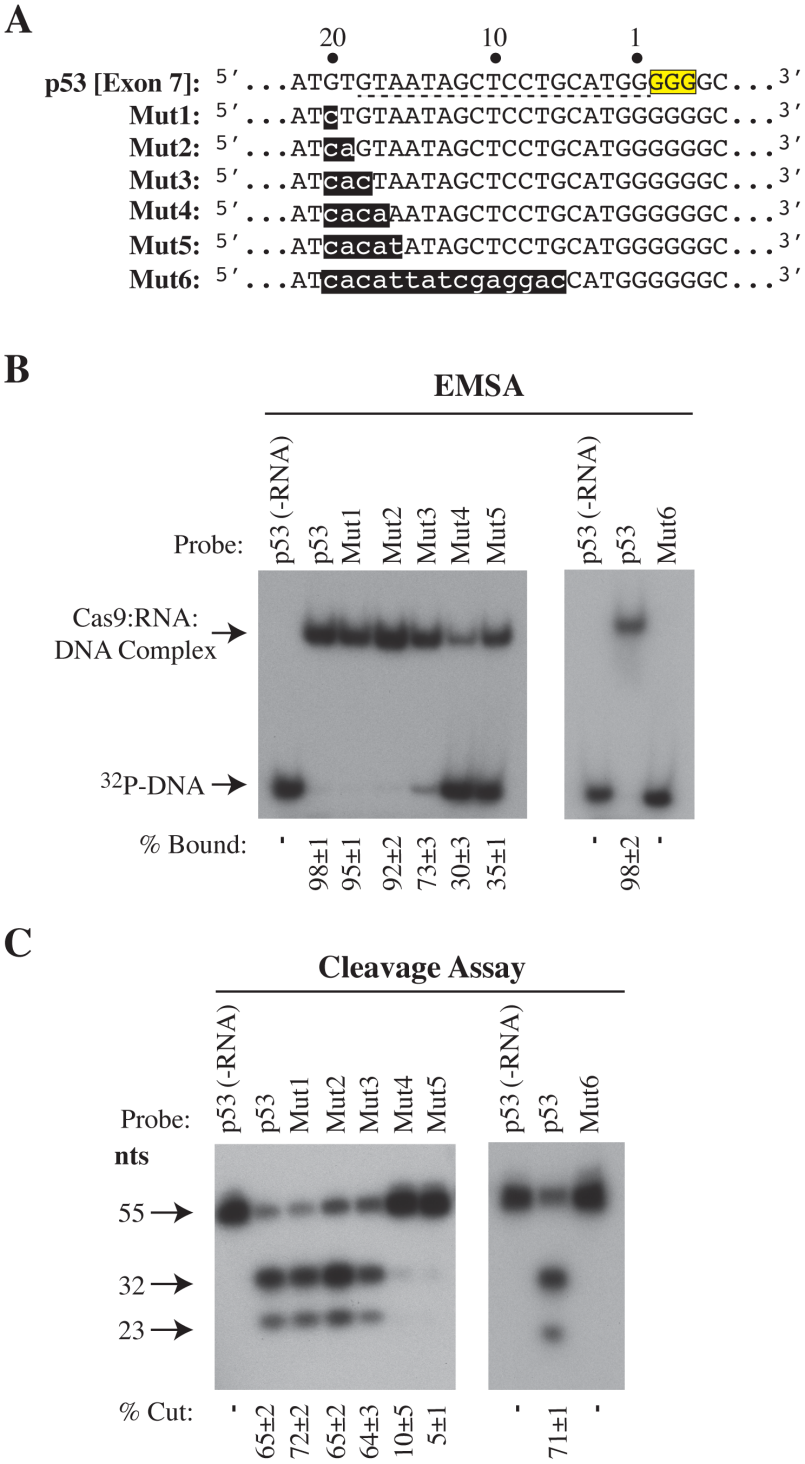
Base Complementarity of the PAM distal target region and the 5′ crRNA end affects engagement of Cas9 nuclease activity. **A.** Sequence comparison of oligonucleotides harboring the wt p53 [Exon 7] target motif (underlined) and mutants harboring mismatches at nucleotides 16–20 of the crRNA guide target. Flanking 5′ and 3′ regions indicated by dots were maintained constant and are the same as in Figure 2A. **B**. Left panel: Assessment of Cas9 binding to oligonucleotides shown in Panel A by EMSA. Right panel: Cleavage reactions of oligonucleotides shown in Panel A. The “-RNA” lanes indicate the absence of crRNA and tracrRNA. Quantifications were performed on a Typhoon Trio Variable Mode Imager with a Fuji imaging screen. n = 3±SD.

We tested this more systematically using a series of mutated target sites spanning the PAM distal sequences of the target (Figure 4A). Mismatches up to and including nucleotide 18 are still efficiently targeted and cleaved by cRNA:tracrRNA activated Cas9, but mismatches that extend to nt 17 or 16 significantly impair cleavage by Cas9 (5–10 fold) while reducing binding only ∼3-fold (Figure 4B). Both activities are eliminated when a crRNA with a seed sequence of only 5 nt is used (Figure 4B). The tolerance of Cas9 binding and cleavage activities for a small number of mismatches in the 5′-distal portion of the target relative to its seed region was further explored by assaying a series of probes containing alternating 2 (Figure S3) or 3 (Figure S4) base pair (bp) mismatches across this area. While binding efficiencies were somewhat consistent for each of the oligonucleotide targets, ranging from 41–62% in reactions containing 2 bp mismatches and 20–38% in reactions containing 3 bp mismatches, defects in cleavage efficiencies were far more varied, ranging from 6–37% and 3–7% in reactions containing 2 bp or 3 bp mismatches, respectively.

**Figure 5:**
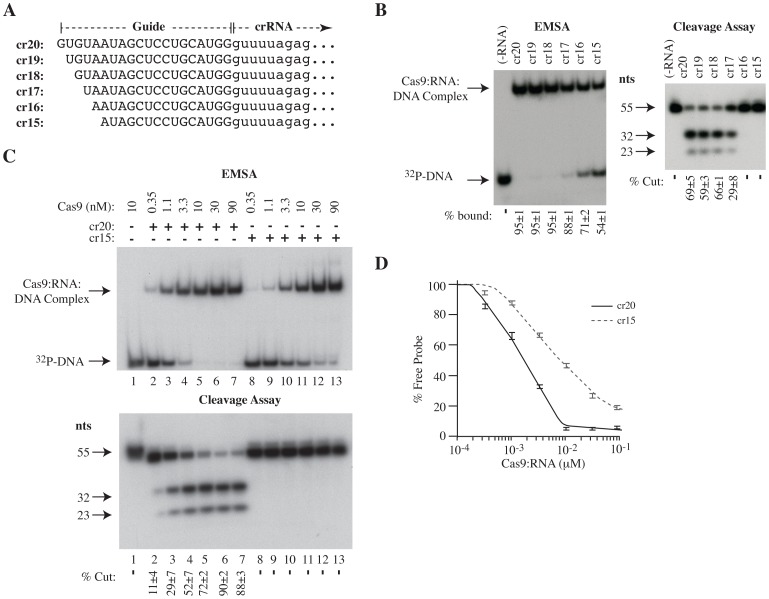
A minimum crRNA guide length of 17 nucleotides is necessary to engage Cas9 target cleavage. **A.** Sequence of crRNA guides of variable length used in the current study. **B**. EMSA (left) and cleavage assay (right) of Cas9/tracrRNA/crRNA combinations using p53 [Exon 7] as probe and resolved on a 5% native polyacrylamide gel and 10% polyacrylamide/8M Urea gel, respectively. The “-RNA” lanes indicate the absence of crRNA and tracrRNA. **C**. EMSA (top) and cleavage assay (bottom) by Cas9 in the presence of p53[Exon 7] target and cr15 or cr20. **D**. Quantitation of Cas9:tracrRNA:cr20:DNA or Cas9:tracrRNA:cr15:DNA complex formation from EMSAs. Quantifications were performed on a Typhoon Trio Variable Mode Imager with a Fuji imaging screen. n = 3±SD.

Notably, these results were reproduced using crRNAs that were progressively truncated from the 5′ end (Figure 5A, B). Although cr15 bound with a 6-fold lower affinity to Cas9:tracrRNA:p53 [Exon7] (K_d_ = 8.8 nM) compared to cr20 (K_d_ = 1.5 nM) (Figure 5C, D and Table 2), this cannot explain the complete absence of target site cleavage, even when the majority of Cas9 is present in a cr15:tracrRNA:DNA complex (Figure 5C, lanes 12 and 13). A minimum length requirement of 17 nts for a crRNA to engage Cas9 nuclease function is consistent with a recent report indicating that 15 nt long “tru-gRNAs” do not mediate genome editing [23].

**Figure 6:**
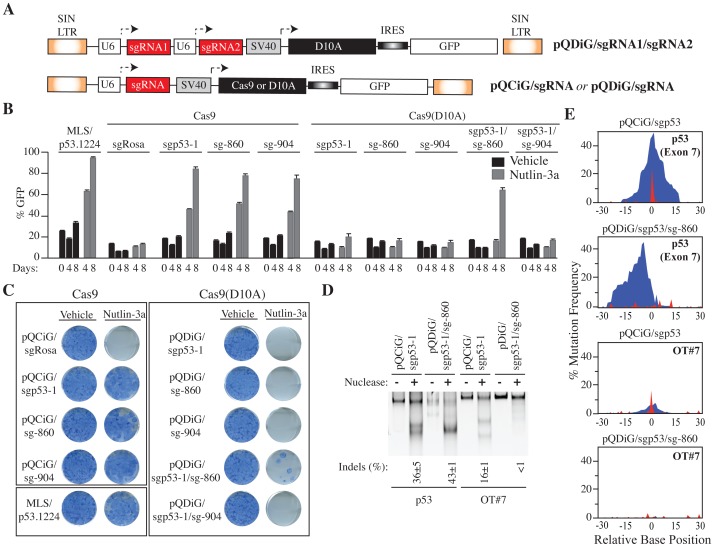
Multiplexed All-in-One vector to Direct Off-set nicking eliminates off-target cleavage. **A.** Schematic representation of retroviral vectors expressing individual or pairs of sgRNAs in the presence of Cas9 or Cas9 (D10A). **B**. Quantitation of GFP^+^ *Arf^−/−^*MEFs transduced with the indicated vectors expressing Cas9 (pQCiG) or Cas9 (D10A) (pQDiG) with individual or pairs of p53 exon 7-targeting sgRNAs (sgp53-1, -860, and -904). The MLS-p53.1224 retrovirus expressing an shRNA to p53 was used as a positive control. Four days after transduction, cells were exposed to vehicle or Nutlin-3a for the indicated period and analyzed on a GUAVA EasyCyte HT flow cytometer (Millipore). n = 3±SD. **C**. Colony formation assay of infected *Arf^−/−^*MEFs with the indicated retroviral vectors. 5000 cells were seeded, exposed to Nutlin-3a for 12 days at which point they were stained with methylene blue. **D**. SURVEYOR assay of p53 [Exon7] and OT#7 from DNA isolated from pQCiG/sgp53-1 and pQDiG/sgp53-1/sg-860 infected cells selected with Nutlin-3a for 12 days. Relative band intensities were quantified using ImageJ (National Institutes of Health). n = 3±SD. **E**. Location and frequency of each sequenced mutation across 60 nucleotides centered around the genomic nucleotide that aligns to 3^rd^ nucleotide of the seed sequence upstream of the PAM of the sgp53-1 guide RNA (the predicted site of Cas9-mediated cleavage), for both *Trp53* (exon 7) and OT#7 loci. *Arf^−/−^*MEFs were transduced with the viruses indicated above the panel. The locus analyzed is indicated on the top right. Blue indicates deletion and red indicates insertions.

This inherent complexity in predicting mismatched off-target cleavage events, due to a combination of position and base composition effects, can be very problematic for some applications (e.g. clinical settings) where even one non-targeted mutation could be deleterious. One way to minimize the issue is to utilize the strategy of “offset nicking”. Here, pairs of sgRNAs directed against a desired locus recruit Cas9 nickases (D10A or H840A mutants) to target the complementary DNA strands to within ∼30 bp of each other [16], [24], [25]. This circumvents off-target mutagenesis since the probability of two mismatched sequences matching both sgRNAs within a short stretch of genomic DNA is very slim. We used this approach to test whether Cas9-driven disruption of the *Trp53* locus via offset nicking would eliminate off-target cleavage at the OT#7 genomic site. Toward this end, we designed multiplexed All-in-One vectors co-expressing 2 sgRNAs from tandemly positioned U6 promoters that target the complementary strand upstream (sg-860) and downstream (sg-904) of the sgp53-1 target region, in conjunction with the Cas9 (D10A) nickase linked to GFP (Figure 6A and Figure S5). We then compared the relative abilities of single versus dual sgRNAs to drive Cas9-mediated genomic editing by positive selection of cells with disrupted endogenous *Trp53* in the presence of Nutlin-3a, a specific inhibitor of the MDM2-p53 interaction [26].

*Arf^−/−^* MEFs were transduced with retroviruses co-expressing Cas9 (pQCiG/x) or Cas9(D10A) nickase (pQCiD/x) with either a single sgRNA (sgp53-1, sg-860 or sg-904) or double sgRNAs (sgp53-1/sg-860 or sgp53-1/sg-904) targeting *Trp53* exon 7; an sgRNA targeting the *Rosa26* locus served as a neutral control (pQCiG/sgRosa), while a previously validated short hairpin construct against p53 (MLS/p53.1224) served as a positive control [27]. Long term expression (14 days) of Cas9 and Cas9(D10A) from the All-in-One vector configuration was well tolerated in *Arf^−/−^* MEFs, as assessed by the absence of changes in either Cas9 protein levels (Figure S6A) or in the GFP^+^ infected cell population over this time period (Figure S6B, C). Enrichment of GFP^+^ cells infected with MLS/p53.1224, pQCiG/sgp53-1, pQCiG/sg-860, pQCiG/sg-904 was observed in the presence of Nutlin-3a but not for vehicle, consistent with Cas9-mediated *Trp53* gene disruption (Figure 6B and Figure S7). This was in contrast to cells infected with the Cas9(D10A) nickase with single sgRNAs (pQDiG/sgp53-1, pQDiG/sg-860, pQDiG/sg-904) whose GFP^+^ proportion remained constant throughout the assay, much like cells infected with the neutral control pQCiG/sgRosa (Figure 6B and Figure S7). Crucially, only cells expressing pQDiG/sgp53-1/sg-860 but not pQDiG/sgp53-1/sg-904 were selected for in the presence of Nutlin-3a indicating mutagenic repair via offset nicking. The lack of enrichment seen with pQDiG/sgp53-1/sg-904 is consistent with 3′ single-stranded overhangs being less efficient substrates than 5′ overhangs for non-homologous end joining (NHEJ) repair [16], [24]. Similar results were obtained in a separate colony-formation assay, where transduced cells were allowed to proliferate over the course of 12 days in the presence of Nutlin-3a or vehicle (Figure 6C). The delayed increase in the proportion of GFP^+^ cells (Figure 6B, compare day 4 to 8) and reduced Nutlin-3a resistance (Figure 6C) for *Arf^−/−^* MEFs infected with pQDiG/sgp53-1/sg-860 relative to pQCiG/sgp53-1 or pQCiG/sg-860 would suggest that offset nicking is less efficient at generating substrates for NHEJ than Cas9, likely due to the necessity for simultaneous complementary strand cleavage and subsequent repair being constrained by the stochastic nature of a sgRNA/Cas9-driven single cleavage event. At the molecular level, we detected indels at the *Trp53* locus for both pQCiG/sgp53-1 and pQDiG/sgp53-1/-860 infected cells, but mutations at the OT#7 allele were only present in pQCiG/sgp53-1 infected cells, as assessed by the Surveyor Assay (Figure 6D) and deep-sequencing of *Trp53* Exon 7 and OT#7 PCR products (Figure 6E). These results highlight the feasibility of using a CRISPR/Cas9 offset nicking “All-in-one” multiplex vector design (bearing 2 sgRNAs and the Cas9(D10A) nickase) to avoid off-target cleavage *in vivo* which maintains reproducible stoichiometry of all the required editing components.

## Discussion

Taken together, our results help broaden our understanding of the mechanism of Cas9 target specificity. Our ChIP-seq data points to widespread binding across diverse genomic regions by Cas9 *in vivo*, far more than previously appreciated, in part due to a much greater tolerance for mismatch base pairing at the 5′ PAM-distal end of the target region. This was mostly revealed through MEME analysis of our sgp53-1/Cas9- and sgp53-3/Cas9-specific enriched peaks which identified a conserved centralized motif that aligned perfectly to the 3′-end of sgRNA target region, reminiscent of a protospacer “seed” sequence requirement (11 nt and 5 nt long sequences for each sgRNA, respectively) (Figure 1). Yet, counterintuitively, even though Cas9 binding seems rampant across the genome, site-specific cleavage is very rare, with what appears to be only a small fraction of the bound mismatched sites acting as substrates for cleavage and subsequent editing events, a result that bodes well for a greater than anticipated target-specificity in applications that use CRISPR/Cas9 genome editing.

Recently, Sharp and colleagues [20] reported on the ChIP-seq analysis for dmCas9 and sgRNAs targeting the Phc1 and Nanog loci. Similar to our data, their study also identified a multitude of off-target sites that could be bound by dmCas9 but again with almost no detectable mutagenic alterations at those very same locations. A majority of their reads followed a much smaller 5 nucleotide “seed” region and, in contrast to our results, they could still detect some binding by dmCas9 to an oligonucleotide that contained only a seed+PAM sequence (but at a much reduced affinity). These small differences are most likely due to the nature of target sequence and variation in mismatch base composition requirements. Kuscu et al. [21] also showed promiscuous binding of Cas9 to many off-target sites (some>1000 depending on the sgRNA tested), but in contrast identified mutations at ∼50% of the sampled sites, albeit at a much lower frequency than at the on-target sites. The elevated mutation rate detected by Kuscu et al. [21] compared to our study and the one from Wu et al. [20] may be due to differences in the amounts of Cas9:sgRNA complex delivered into the cells; we generally aim to infect cells at an MOI of ∼1 which results in much reduced level of Cas9:sgRNA expression relative to conventional transfection protocols.

We note that the *Trp53* pseudogene, which differs by only 2 nucleotides from the wild-type *Trp53* [Exon 7] target site (C to T at the 8^th^ nt and A to G at the 15^th^ nt) was not detected in our sgp53-1 ChIP-seq data, consistent with the absence of editing that we previously documented at this locus with sgp53-1 [22]. Yet when tested *in vitro*, an oligonucleotide harboring the *Trp53* pseudogene target sequence was a substrate for cleavage by Cas9 (R.C., data not shown). One possible explanation for this discrepancy is if the *Trp53* pseudogene is inaccessible to Cas9 *in vivo* due to epigenetic heterochromatin packing, as was recently suggested to negatively impact Cas9:DNA interaction *in vivo* [20].

**Figure 7:**
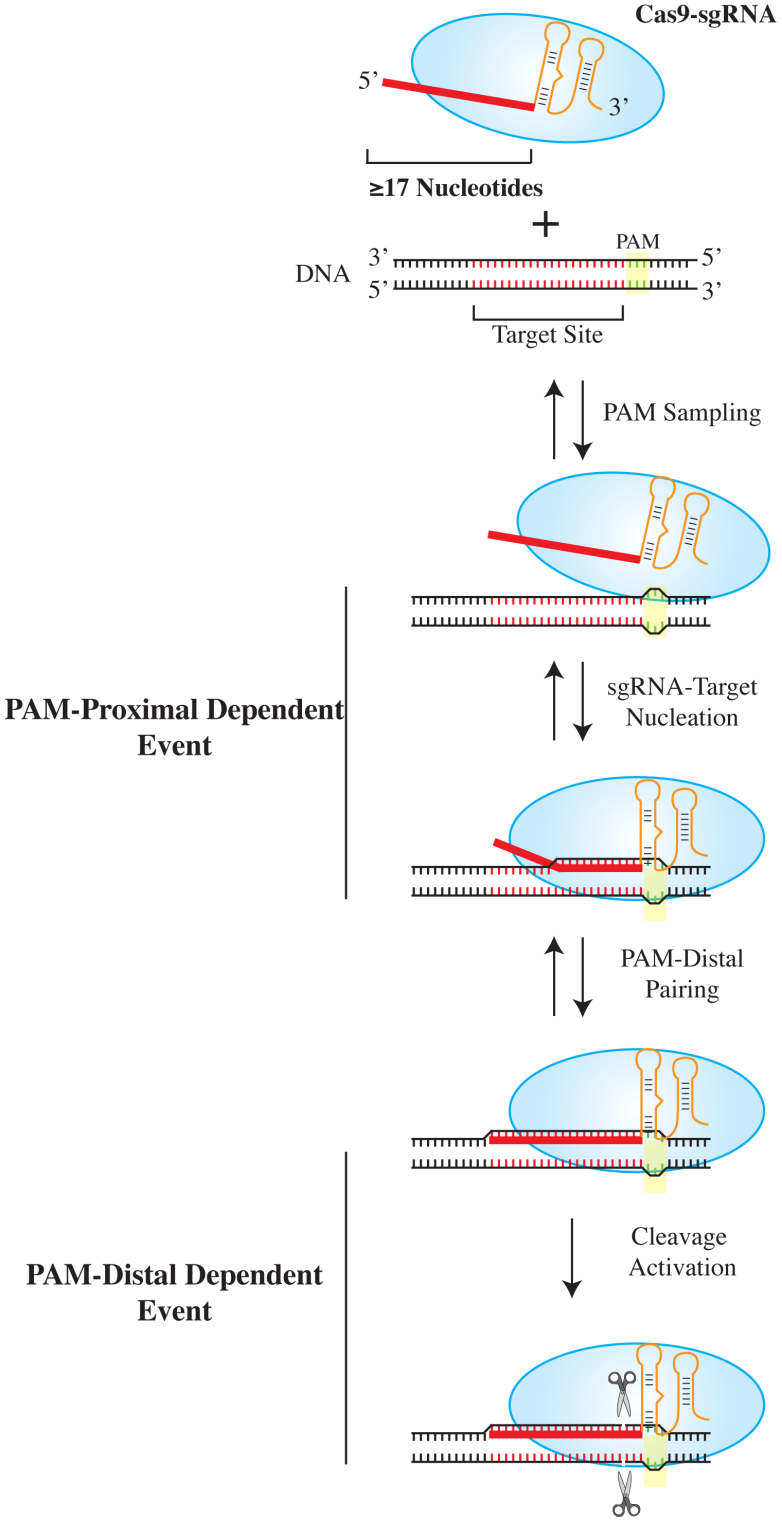
Model Illustrating Target Sequence Dependency for Cas9 Target Binding and Cleavage. Following PAM sampling by the Cas9:sgRNA complex, only PAM-proximal sequences are required for Cas9:sgRNA:target nucleation and are sufficient for complex formation [11]. Our study indicates that PAM-distal complementarity with the sgRNA (and a minimum sgRNA guide length of 17 nucleotides) is required to engage Cas9 target cleavage.

Nevertheless, our results suggest that while a few mismatches are tolerated in the PAM-distal region of a target sequence, more extensive mismatches result in considerably poorer substrates. In support of this, and most strikingly, a truncated crRNA of 16 nucleotides completely abolished all detectable target cleavage (Figure 5). Perhaps involved in this interaction is the Tyr1013 residue which, based on recent crystal structure data, appears to stack with the 5′ most guanosine of the sgRNA in Cas9 [28]. Tyr1013 resides within a flexible loop (T.M.S. and J.P., unpublished data) and might relay information to Cas9 via a conformation change associated with the sgRNA 5′- end and PAM distal target recognition. Taken together, we propose a bifunctional sequence requirement for full Cas9 activity (Figure 7). While crRNA base-pairing in PAM-proximal region is necessary for seeding of the R-loop between Cas9 and the DNA target, as described by Sternberg et al. [11], our *in vitro* and *in vivo* data indicates this to be insufficient for complete endonucleocytic activity. Rather, the extent of base complementarity between PAM-distal target and 5′ crRNA sequences is what fully determines cleavage activity. This elaborate multistep mechanism adopted by the CRISPR/Cas9 bacterial host-defense systems may have evolved as a mechanism to allow for target site sampling while minimizing cleavage within host genomes.

## Materials and Methods

### Reagents

Nutlin-3a was obtained from Sigma, dissolved in 100% DMSO, and stored at −20°C. Nutlin-3a was used at a final concentration of 5 µM. Antibodies used for Western blots in this study were directed against FLAG tag (M2; Sigma) and eEF2 (#2332; Cell Signaling).

### Cell Culture and Lentiviral/Retroviral Infections

*Arf^−/−^* MEFs (a kind gift of Dr. Scott Lowe [Memorial Sloan Kettering, NY]) [29] were maintained in DMEM supplemented with 10% fetal bovine serum, 100 U/ml PEN/STP, and 100 U/ml Gln. All retroviral packaging was performed using ecotropic Phoenix cells (obtained from the ATCC) according to established protocols (http://www.stanford.edu/group/nolan/retroviral_systems/retsys.html). The pQCiG/sgRosa and sgp53 retroviral constructs used in this study have been previously described [22]. MLS/p53.1224, an MSCV- based retrovirus expressing an shRNA against p53, has been previously characterized [27].

The following sgRNAs were used in this study, with the guide sequence provided in parenthesis: sgp53-1 [^5′^ GUGUAAUAGCUCCUGCAUGG ^3′^], sgp53-3 (previously p53(b) [22]) [^5′^ GAAGUCACAGCACAUGACGG ^3′^], TLR [^5′^ GAGCAGCGUCUUCGAGAGUG ^3′^], Rosa26 [^5′^ GAAGAUGGGCGGGAGUCUUC ^3′^], sg-860 [^5′^ CUAUUACACAUGUACUUGUAG ^3′^], and sg-904 [^5′^ GAUGGUAAGGAUAGGUCGG ^3′^].

### ChIP-seq and Motif Discovery

*Arf^−/−^* MEFs infected with pQdmCiG/sgp53-1 or pQdmCiG/sgp53-3 were further enriched for GFP expression via flow cytometry and used in ChIP-seq experiments. Chromatin immunoprecipitations were conducted as previously described using an anti-FLAG antibody (F1804, Sigma) [30]. Immunoprecipitated DNA was used to generate ChIP-seq libraries using barcode adaptors from the NEBNext ChIP-seq Library Prep Reagent Set for Illumina (E6200S, New England Biolabs) [31]. The ChIP-seq libraries were quantified using the Quant-iT PicoGreen dsDNA Assay Kit (P11496, Life Technologies) and 51 base single-end reads were obtained on an Illumina HiSeq 2500 unit (TCGA Facility, Centre for Applied Genomics, The Hospital for Sick Children, Toronto).

The FASTX-toolkit (http://hannonlab.cshl.edu/fastx_toolkit/) was used for barcode splitting. After barcode trimming, sequence reads were mapped to the mouse genome (mm9) using Bowtie 2 (version 2.0.2) [32]. Duplicate reads were removed using Picard (version 1.69, URL: http://picard.sourceforge.net) and peak calling was performed using HOMER (http://biowhat.ucsd.edu/homer/chipseq/). Peaks overlapping with the blacklisted genomic regions (mm9) were filtered out (ENCODE blacklisted region URL: https://sites.google.com/site/anshulkundaje/projects/blacklists). To maintain a false discovery rate (FDR) of below 0.1%, a threshold peak value of >10.53 (sgp53-1/dmCas9 normalized read count per 10^7^ reads), >16.61 (sgp53-3/dmCas9 normalized read count per 10^7^ reads), and <9.88 (dmCas9 normalized read count per 10^7^ reads) was used. A 4-fold difference between the read density of the sample and the matched WCE was also required to call a true peak. Sequences from each peak region (±150 bps from the center position) were extracted and used for *de novo* motif analysis using MEME-ChIP tools [33]. Identified seed sequences and adjacent PAM were used for CentriMo determination.

### Ion Torrent Sequencing

Selected genomic regions flanking p53 and predicted Cas9 target sites (Table S3) were amplified using bar coded primers with engineered adaptor regions. Off-target loci (with the exception of OT#7/p53-1 and Peak_33/p53-3) were amplified by PCR using Phusion HiFi polymerase (NEB) with 5 cycles of annealing temperature at 56°C followed by 20 cycles with an annealing temperature of 68°C. To amplify off-target locus #7 and Peak_33 (p53-3), a touch down PCR protocol with Phusion HiFi polymerase (NEB) was used. The annealing temperature was reduced from 68°C to 56°C with a 1°C decrease/cycle over 13 cycles, followed by 15 cycles with an annealing temperature at 56°C. PCR products were purified using Ampure XP beads (Beckman). DNA samples were quantified using the Quant-iT PicoGreen dsDNA Assay Kit (P11496, Life Technologies) and pooled in equimolar ratios. Sequencing libraries were then sequenced on an Ion Torrent Personal Genome Machine (PGM) as recommended by the manufacturer (Life Technologies). The average background mutation rate for all 43 loci analyzed in control, pQCiG/sgRosa infected *Arf^−/−^* MEFs was 0.3%.

### Electrophoretic Gel Shift and *In Vitro* Cleavage Assays

Vectors pMJ806 and pMJ841 expressing Cas9 and dmCas9, respectively were obtained from Addgene (https://www.addgene.org/). Recombinant wild-type (wt) and double mutant (dm) (D10A/H840A) Cas9 protein were purified as previously described [2]. TracrRNA was produced by *in vitro* transcription from a PCR-amplified template harboring the tracrRNA sequence downstream of an embedded T7 promoter (template sequence: ^5′^ TAATACGACTCACTATAGGGGACAGCATAGCAAGTTAAAATAAGGCTAGTCCGTTATCAACTTGAAAAAGTGGCACCGAGTCGGTGCTTTTT ^3′^ [T7 promoter is underlined]). The PCR primers used to amplify the template sequence were PCR-F primer (^5′^ GAGGATTAATACGACTCACTATAGGGGAC ^3′^) and PCR-R primer (^5′^ AAAAAGCACCGACTCGGTGCC ^3′^). All crRNAs were purchased from Integrated DNA Technology Inc. (Coralville, IA) and were HPLC purified. DNA target oligonucleotides were ordered from Integrated DNA Technology Inc. (Coralville, IA) and the two strands annealed and gel purified as described [11]. The target duplexes were kinased using T4 polynucleotide kinase (New England Biolabs, Beverly, MA, USA) and γ-^32^P-ATP (Perkin-Elmer, Waltham, MA, USA), followed by spin column purification (Bio Basic Inc., Ontario, Canada). Gel shifts and *in vitro* cleavage assays were performed as described [11] using 1 nM radiolabeled dsDNA target, 10 nM recombinant Cas9 (unless otherwise stated), 20 nM tracrRNA, and 20 nM crRNA. All reactions contained 2.5 µg/ml heparin. Complexes were resolved on 5% native polyacrylamide gels (19∶1; acrylamide∶bisacrylamide). For K_D_ determinations, the fraction of RNA bound at each Cas9 concentration was determined and the data fit with a standard binding isotherm using Kaleidograph (Synergy Software).

Visualization of p53 [Exon 7] cleavage products by Cas9/tracr/crRNA was achieved on 10% polyacrylamide/8M urea gels (19∶1; acrylamide∶bisacrylamide). In some cases, Cas9:RNA:DNA complexes were purified from EMSA polyacrylamide gels. Here, complexes were excised from the 5% native polyacrylamide gels using a razor blade, treated with 20 µg PK in 200 µl PK buffer (100 mM Tris-HCl _[pH 7.5]_, 12.5 mM EDTA, 150 mM NaCl, 1%SDS) for 1 h at 55°C. Following incubation, samples were centrifuged for 15 min at 14,000×g and the supernatant phenol/chloroform extracted, followed by ethanol precipitation of the nucleic acid in the presence of 1 µg glycogen (Ambion) as carrier at −20°C. DNA was recovered by centrifugation (14,000×g for 15 mins) and resolved on a 10% polyacrylamide/8 M urea gel.

### SURVEYOR assay

The SURVEYOR assay was performed as described previously [22]. Briefly, genomic DNA was prepared from samples exposed to Nutlin-3a using standard methods. The p53 target and OT#7 loci were amplified using primers (^5′^ TTCACCTGGATCCTGTGTCT ^3′^ and ^5′^ TTCACCTGGATCCTGTGTCT ^3′^ for p53 and ^5′^ GGTGCAATCCTCAGAAGAAG ^3′^ and ^5′^ TGACTAGATGCTATATGTGC ^3′^ for OT#7) and PCR conditions as described [22]. Mutations in the amplicons were assessed using the SURVEYOR mutation detection kit according to the manufacturer′s instructions (Transgenomic) and reactions analyzed on 10% non-denaturing polyacrylamide gels. Relative band intensities were quantified using ImageJ (National Institutes of Health).

## Supporting Information

Figure S1 **Relative Cas9 and dmCas9 expression in transduced** ***Arf^−/−^*** **MEFs.** Western blot of lysates from uninfected (lane 1) or *Arf^−/−^* MEFs infected with pQCiG/sgp53-1, pQdmCiG, or pQdmCiG/sgp53-1 (lanes 2–4) and used for mutation probing (see Figure 1E) or in ChIP-seq experiments (see Table 1). eEF2 was used as a loading control.
(TIF)

Figure S2 **Assessment of DNA cleavage within Cas9:RNA:DNA complexes resolved by EMSA.** **A**. Assessment of Cas9 binding (2 pmoles) to oligonucleotide probes shown in Figure 3A. Reactions were resolved on a 5% native polyacrylamide gel. Quantifications were performed on a Typhoon Trio Variable Mode Imager with a Fuji imaging screen. n = 2±Error of the Mean. **B**. Assessment of cleaved products isolated from the Cas9:RNA:DNA complexes resolved in Panel A. EMSA complexes from panel A (highlighted by a box) were purified as described in the Materials and Methods and resolved on a 10% polyacrylamide/8 M urea gel.
(TIF)

Figure S3 **Base Complementarity of the PAM distal target region and the 5′ crRNA end affects licensing of Cas9 endonucleolytic activity.** **A**. Oligonucleotide set used to document consequences of mismatches within PAM distal sequences on target recognition and cleavage. Oligonucleotides harboring two mismatches at nucleotides 14–19 of the p53 guide target. The PAM is highlighted in yellow. Mismatches were chosen to maintain the purine/pyrimidine ratio and are highlighted in blue. **B**. Assessment of Cas9 binding and cleavage of oligonucleotides harboring two mismatches between nucleotides 14–19 of the p53 guide target. EMSA complexes and cleavage reactions were resolved on polyacrylamide gels and quantitated. n = 7±SD.
(TIF)

Figure S4 **Mismatches within the PAM-distal region affect licensing of Cas9 endonucleolytic activity.** **A**. Sequence comparison of oligonucleotides harboring the WT p53 [Exon 7] target motif (underlined) with adjacent PAM (highlighted in yellow) and mutants harboring 3 mismatches at nucleotides 14–19 of the target DNA (highlighted in black). Flanking 5′ and 3′ regions indicated by dots were maintained constant and are the same as in Figure 2A. **B**. Left panel: Assessment of Cas9 binding to oligonucleotides shown in Panel A by EMSA. Right panel: Cleavage reactions of oligonucleotides shown in Panel A. The “-RNA” lanes indicate the absence of crRNA and tracrRNA. Quantifications were performed on a Typhoon Trio Variable Mode Imager with a Fuji imaging screen. n = 2±Error of the mean.
(TIF)

Figure S5 **Off-set nicking strategy at p53 Exon 7.** An expanded view of exon 7 is shown with PAM motifs highlighted in yellow and sequences corresponding to the sgRNAs highlighted by a black line. In the presence of Cas9 (D10A), the combination of sgp53-1 and sg-860 is predicted to generate 5′ single strand overhangs (boundaries denoted by upward and downward arrows). The combination of sgp53-1 and sg-904 is predicted to generate 3′ single strand overhangs (boundaries denoted by upward and downward arrows).
(TIF)

Figure S6 **Ectopic expression of Cas9 and Cas9(D10A) in** ***Arf^−/−^*** **MEFs is well tolerated.** **A**. *Arf^−/−^*MEFs were infected with All-in-One vectors encoding Cas9 (pQCiG) or Cas9(D10A) (pQDiG) and individual sgRNAs or pairs of p53 exon 7 targeting sgRNAs (sgp53-1 and -860). Four (t = 0) and 18 (t = 14) days after transduction, cells were harvested and Western Blot analyses performed on whole cell extracts probing for the relative levels of Cas9. eEF2 was used as a loading control. **B**. Representative experiment of an analysis by flow cytometry of *Arf^−/−^*MEFs transduced with the indicated retroviral constructs. Four days after transduction (t = 0), cells were analyzed on a GUAVA EasyCyte HT flow cytometer (Millipore). Cells were maintained in culture for an addition 14 days at which point they were re-analyzed. The percent GFP^+^ cells is denoted. **C**. Quantitation of GFP^+^ *Arf^−/−^*MEFs transduced cells with the indicated vectors at the denoted time points. n = 3; error bars denote SD.
(TIF)

Figure S7 **Representative flow cytometry analysis of** ***Arf^−/−^*** **MEFs transduced with the indicated retroviral constructs.** *Arf^−/−^*MEFs expressing Cas9 (pQCiG) or Cas9(D10A) (pQDiG) and single sgRNAs or pairs of p53 exon 7 targeting sgRNAs (sgp53-1, -860, and -904). The MLS/p53.1224 retrovirus expressing an shRNA to p53 was included as a positive control. Four days after transduction, cells were exposed to vehicle or Nutlin-3a and analyzed at the indicated time points on a GUAVA EasyCyte HT flow cytometer (Millipore). The percent GFP^+^ cells is denoted.
(TIF)

Table S1 **ChIP-Seq peaks ranked by read counts and enriched in sgp53-1/dmCas9 and sgp53-3/dmCas9 immunoprecipitates relative to dmCas9 immunoprecipitates.**
(XLSX)

Table S2 **Chromosome Location of Sites harboring 5′CTCCTGCATGG3′+PAM or 5′GCACATGACGG3′+PAM motif.**
(XLSX)

Table S3 **Genomic loci probed for mutational analysis.** PEAK IDs harboring and de novo predicted sites harboring a motif correspond to that identified by ChIP-Seq of sgp53-1/dmCas9 or sgp53-3/dmCas9.
(XLSX)
