# Supplementary figures and images for: Protospacer Adjacent Motif (PAM)-Distal Sequences Engage CRISPR Cas9 DNA Target Cleavage
Source: PLoS One. 2014 Oct 2;9(10):e109213. doi: 10.1371/journal.pone.0109213 (PMC4183563; doi:10.1371/journal.pone.0109213)

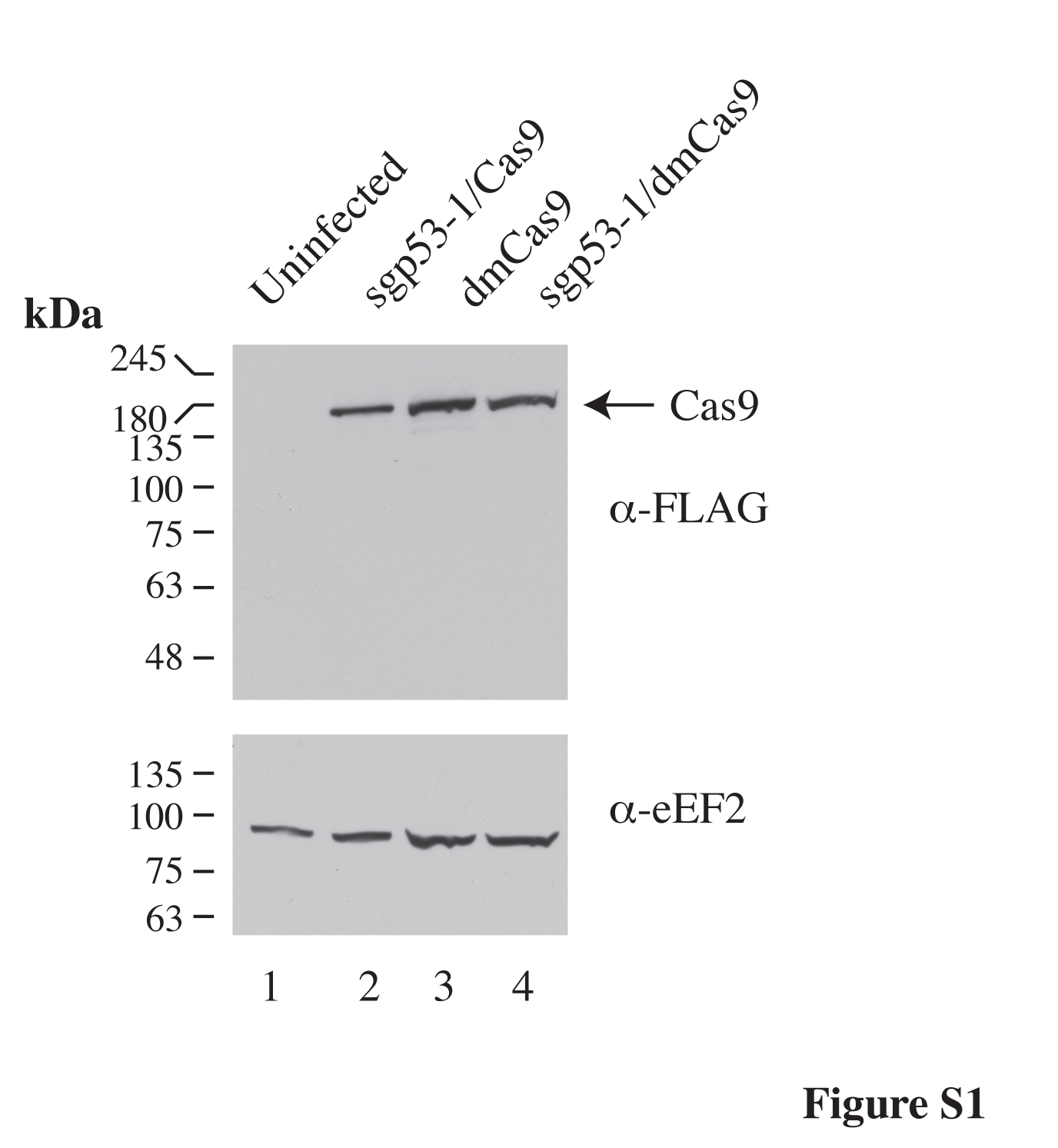

Supplement: Figure S1 — Relative Cas9 and dmCas9 expression in transduced Arf−/− MEFs. Western blot of lysates from uninfected (lane 1) or Arf−/− MEFs infected with pQCiG/sgp53-1, pQdmCiG, or pQdmCiG/sgp53-1 (lanes 2–4) and used for mutation probing (see Figure 1E) or in ChIP-seq experiments (see Table 1). eEF2 was used as a loading control. (TIF) [file pone.0109213.s001.tif]

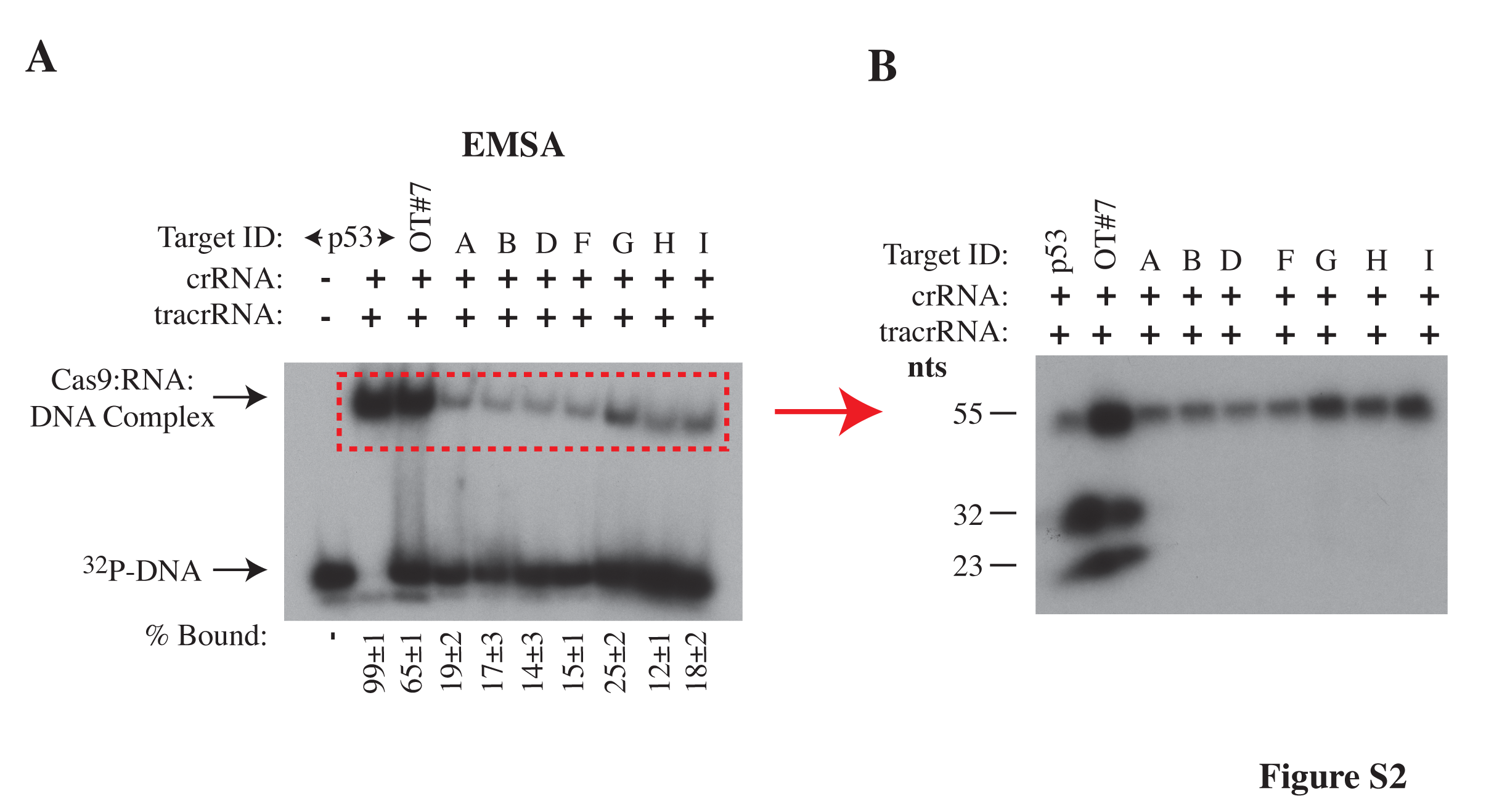

Supplement: Figure S2 — Assessment of DNA cleavage within Cas9:RNA:DNA complexes resolved by EMSA. A. Assessment of Cas9 binding (2 pmoles) to oligonucleotide probes shown in Figure 3A. Reactions were resolved on a 5% native polyacrylamide gel. Quantifications were performed on a Typhoon Trio Variable Mode Imager with a Fuji imaging screen. n = 2±Error of the Mean. B. Assessment of cleaved products isolated from the Cas9:RNA:DNA complexes resolved in Panel A. EMSA complexes from panel A (highlighted by a box) were purified as described in the Materials and Methods and resolved on a 10% polyacrylamide/8 M urea gel. (TIF) [file pone.0109213.s002.tif]

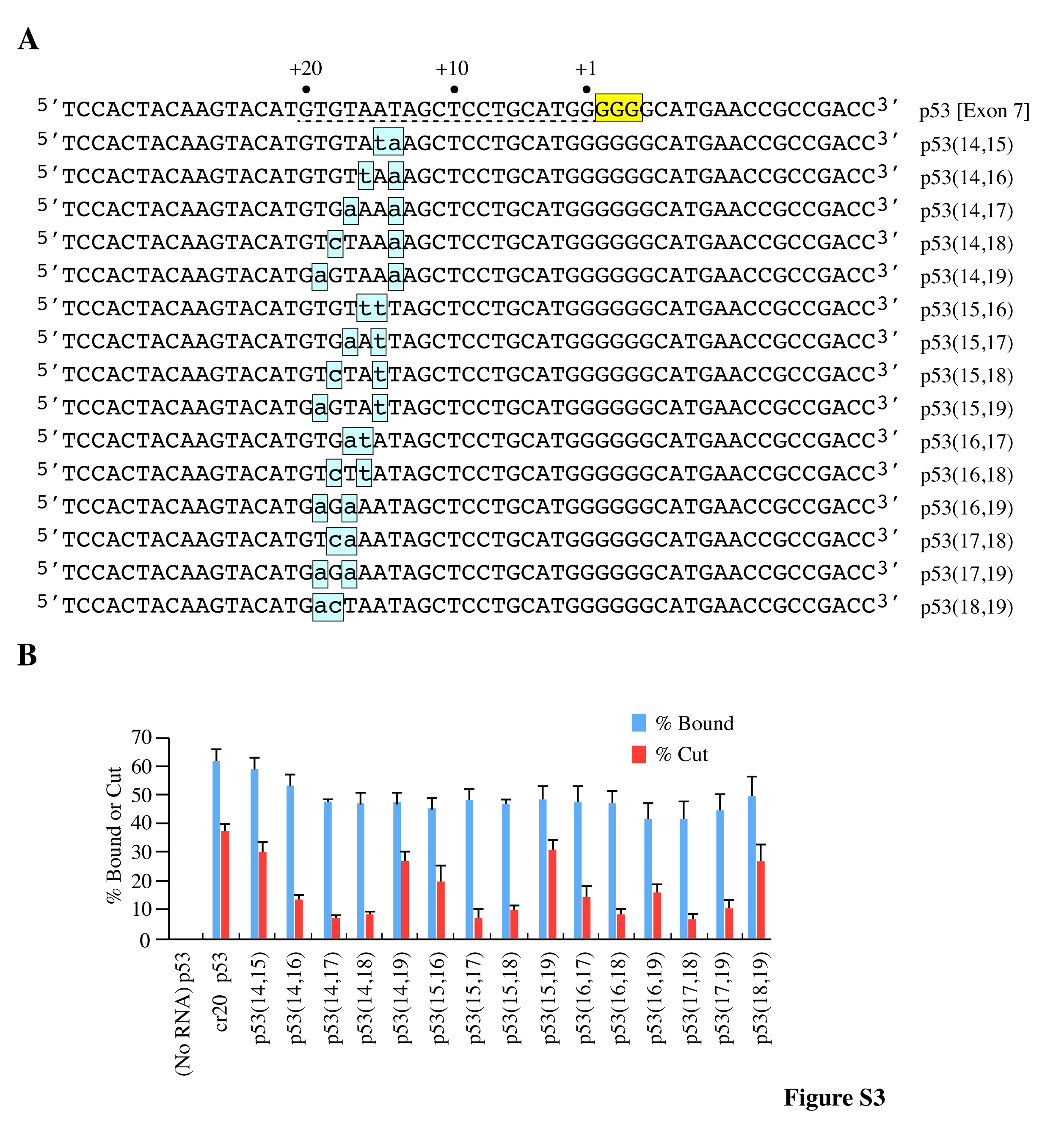

Supplement: Figure S3 — Base Complementarity of the PAM distal target region and the 5′ crRNA end affects licensing of Cas9 endonucleolytic activity. A. Oligonucleotide set used to document consequences of mismatches within PAM distal sequences on target recognition and cleavage. Oligonucleotides harboring two mismatches at nucleotides 14–19 of the p53 guide target. The PAM is highlighted in yellow. Mismatches were chosen to maintain the purine/pyrimidine ratio and are highlighted in blue. B. Assessment of Cas9 binding and cleavage of oligonucleotides harboring two mismatches between nucleotides 14–19 of the p53 guide target. EMSA complexes and cleavage reactions were resolved on polyacrylamide gels and quantitated. n = 7±SD. (TIF) [file pone.0109213.s003.tif]

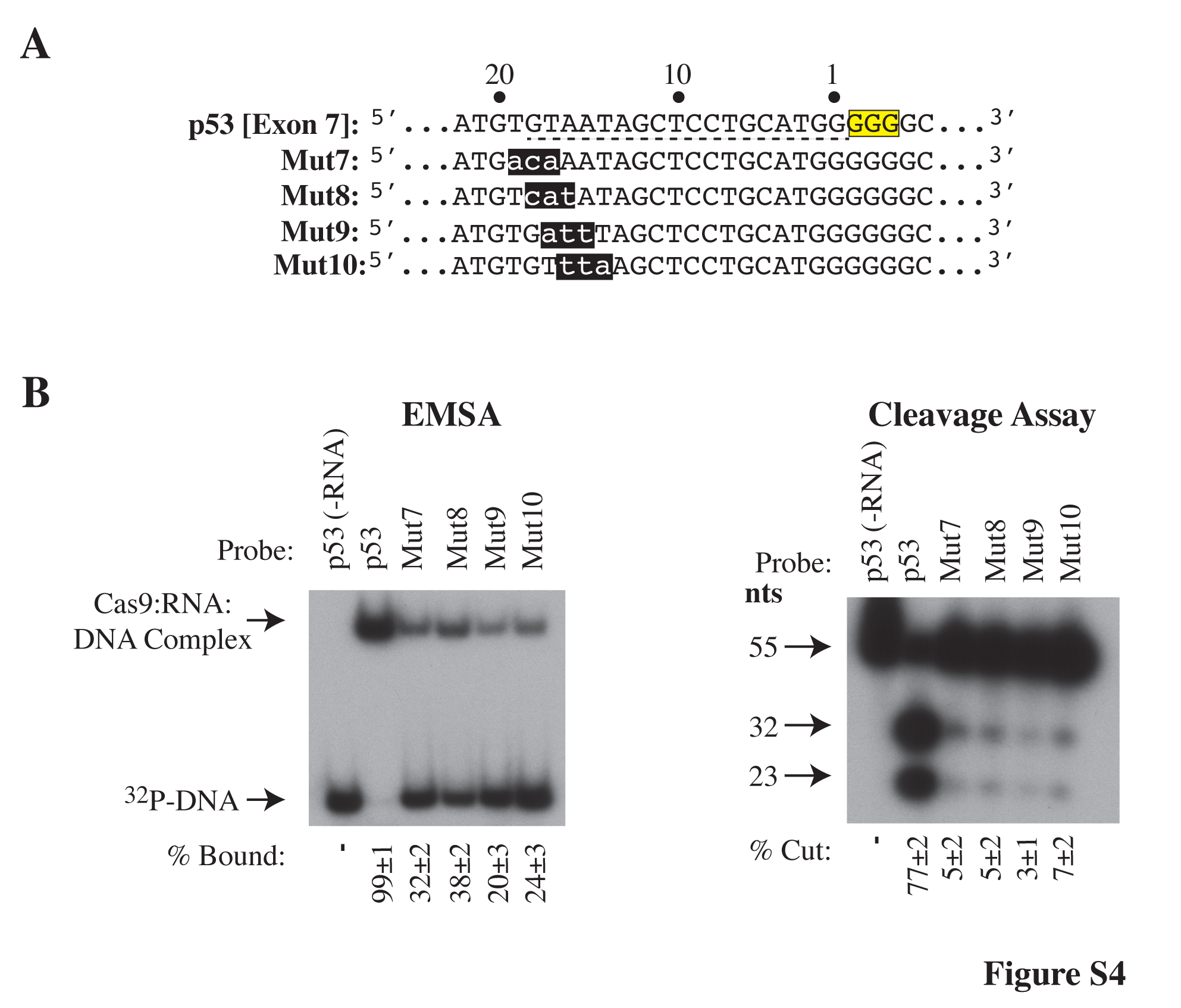

Supplement: Figure S4 — Mismatches within the PAM-distal region affect licensing of Cas9 endonucleolytic activity. A. Sequence comparison of oligonucleotides harboring the WT p53 [Exon 7] target motif (underlined) with adjacent PAM (highlighted in yellow) and mutants harboring 3 mismatches at nucleotides 14–19 of the target DNA (highlighted in black). Flanking 5′ and 3′ regions indicated by dots were maintained constant and are the same as in Figure 2A. B. Left panel: Assessment of Cas9 binding to oligonucleotides shown in Panel A by EMSA. Right panel: Cleavage reactions of oligonucleotides shown in Panel A. The “-RNA” lanes indicate the absence of crRNA and tracrRNA. Quantifications were performed on a Typhoon Trio Variable Mode Imager with a Fuji imaging screen. n = 2±Error of the mean. (TIF) [file pone.0109213.s004.tif]

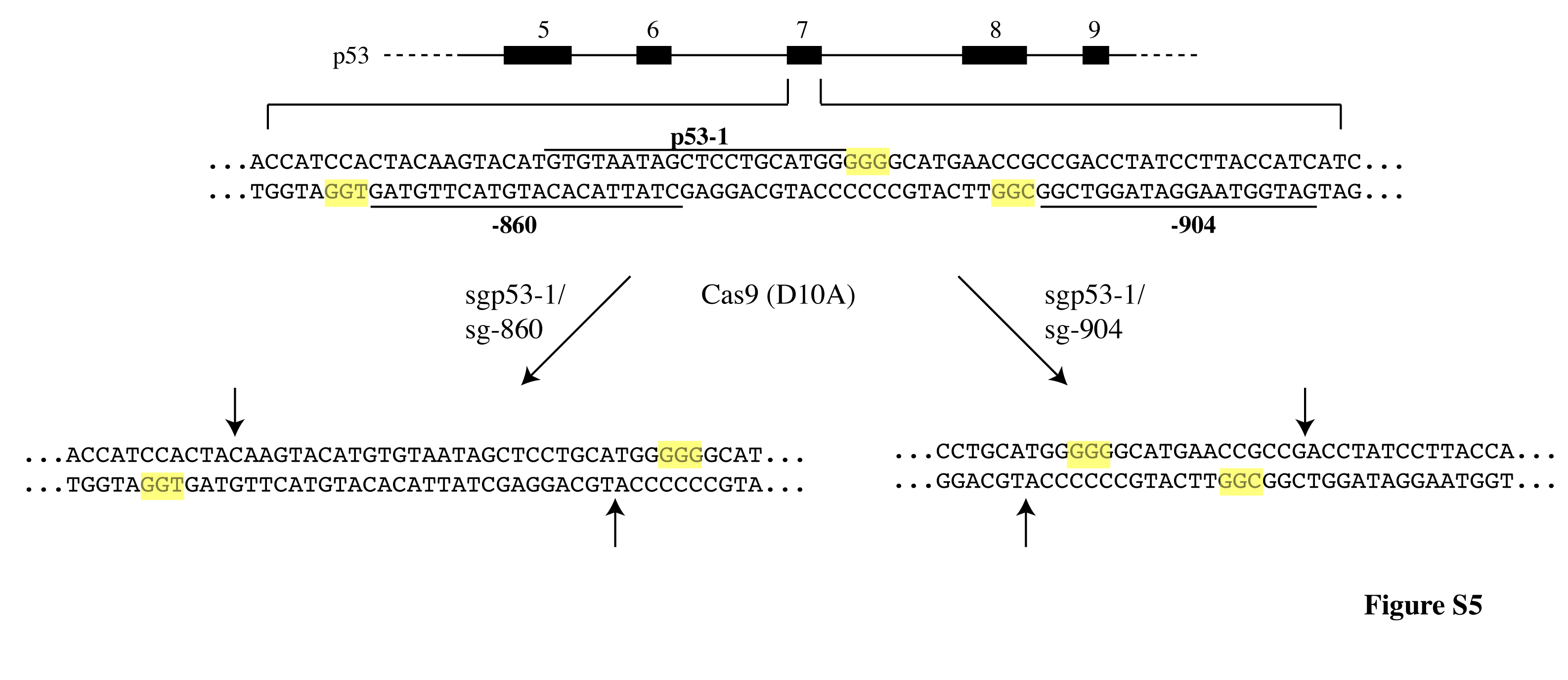

Supplement: Figure S5 — Off-set nicking strategy at p53 Exon 7. An expanded view of exon 7 is shown with PAM motifs highlighted in yellow and sequences corresponding to the sgRNAs highlighted by a black line. In the presence of Cas9 (D10A), the combination of sgp53-1 and sg-860 is predicted to generate 5′ single strand overhangs (boundaries denoted by upward and downward arrows). The combination of sgp53-1 and sg-904 is predicted to generate 3′ single strand overhangs (boundaries denoted by upward and downward arrows). (TIF) [file pone.0109213.s005.tif]

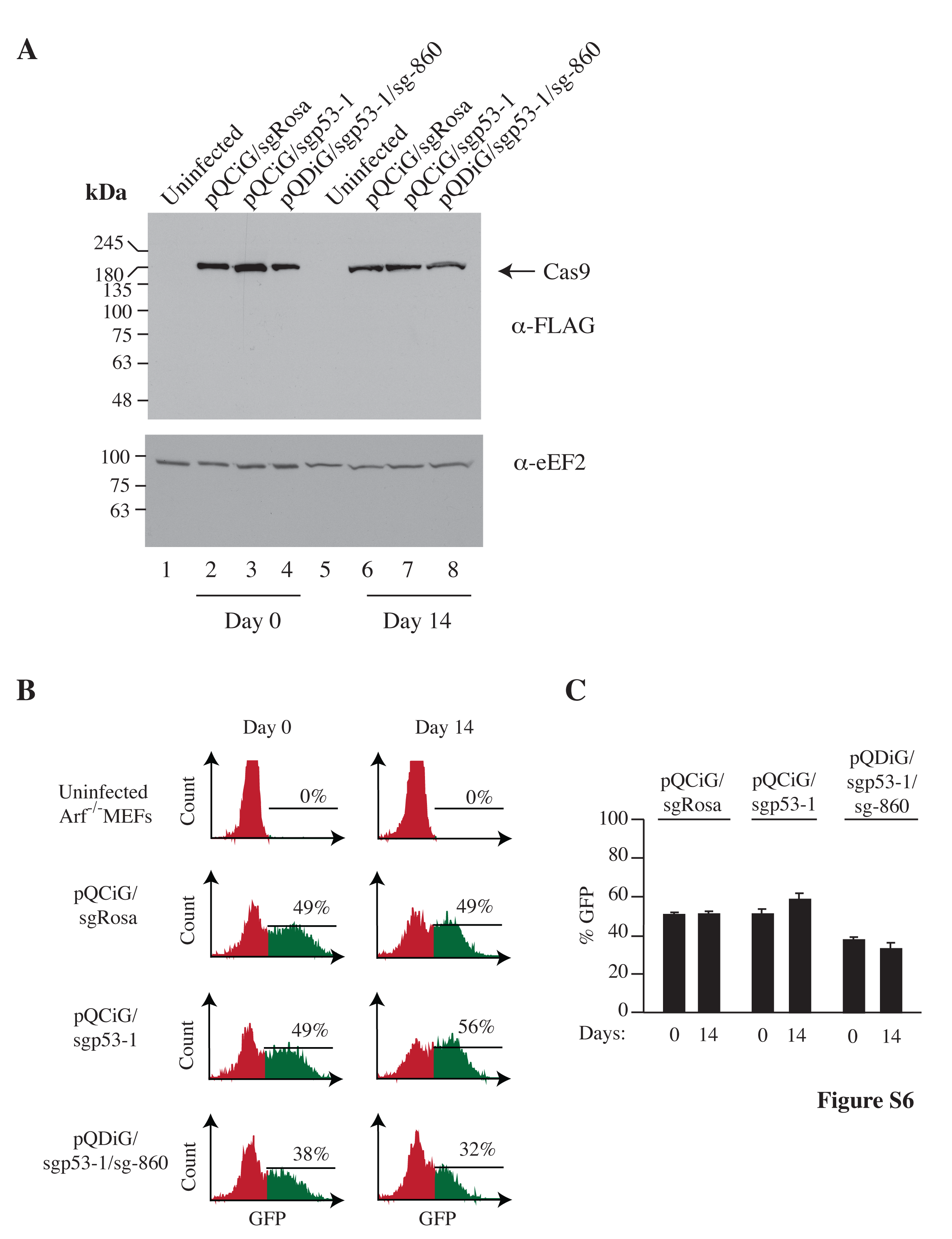

Supplement: Figure S6 — Ectopic expression of Cas9 and Cas9(D10A) in Arf−/− MEFs is well tolerated. A. Arf−/−MEFs were infected with All-in-One vectors encoding Cas9 (pQCiG) or Cas9(D10A) (pQDiG) and individual sgRNAs or pairs of p53 exon 7 targeting sgRNAs (sgp53-1 and -860). Four (t = 0) and 18 (t = 14) days after transduction, cells were harvested and Western Blot analyses performed on whole cell extracts probing for the relative levels of Cas9. eEF2 was used as a loading control. B. Representative experiment of an analysis by flow cytometry of Arf−/−MEFs transduced with the indicated retroviral constructs. Four days after transduction (t = 0), cells were analyzed on a GUAVA EasyCyte HT flow cytometer (Millipore). Cells were maintained in culture for an addition 14 days at which point they were re-analyzed. The percent GFP+ cells is denoted. C. Quantitation of GFP+ Arf−/−MEFs transduced cells with the indicated vectors at the denoted time points. n = 3; error bars denote SD. (TIF) [file pone.0109213.s006.tif]

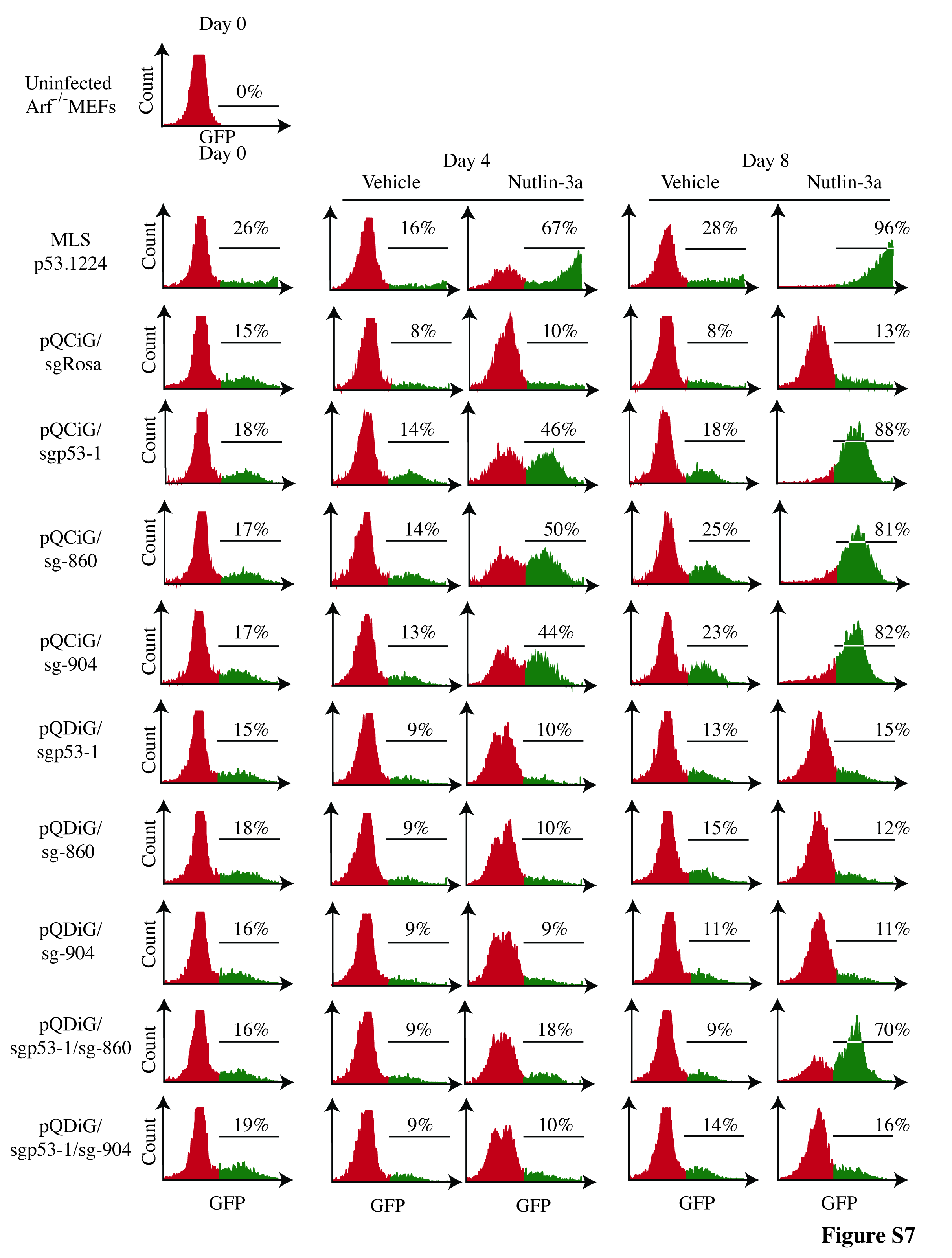

Supplement: Figure S7 — Representative flow cytometry analysis of Arf−/− MEFs transduced with the indicated retroviral constructs. Arf−/−MEFs expressing Cas9 (pQCiG) or Cas9(D10A) (pQDiG) and single sgRNAs or pairs of p53 exon 7 targeting sgRNAs (sgp53-1, -860, and -904). The MLS/p53.1224 retrovirus expressing an shRNA to p53 was included as a positive control. Four days after transduction, cells were exposed to vehicle or Nutlin-3a and analyzed at the indicated time points on a GUAVA EasyCyte HT flow cytometer (Millipore). The percent GFP+ cells is denoted. (TIF) [file pone.0109213.s007.tif]
